# Well-Being Profiles of Family Caregivers of Patients With Dementia From Romania: A Latent Profile Analysis

**DOI:** 10.7759/cureus.83103

**Published:** 2025-04-27

**Authors:** Liviu Florian Tatomirescu, Gabriel-Ioan Prada, Cristiana Susana Glavce, Richard David-Rus, Adriana Borosanu

**Affiliations:** 1 Department of Psychiatry, C.F.2 Clinical Hospital, Bucharest, ROU; 2 Clinical Department IV, National Institute of Gerontology and Geriatrics Ana Aslan, Bucharest, ROU; 3 Department of Biomedical Anthropology, Institute of Anthropology Francisc I. Rainer, Romanian Academy, Bucharest, ROU

**Keywords:** dementia patients, health of family caregivers, lpa analysis, ryff’s scale, well-being

## Abstract

Introduction: The rising prevalence of dementia has increased the demand for long-term care, with family members often assuming caregiving responsibilities. While this form of care reduces healthcare costs and improves patients' quality of life, it also exposes caregivers to physical and mental health challenges, often rendering them "invisible patients." Well-being remains a key focus in both medical and psychosocial research and can be assessed through Ryff’s eudaimonic framework. This study aims to identify distinct latent profiles of family caregivers based on well-being patterns, highlighting, through a person-centered approach, the vulnerabilities and resources associated with each profile.

Methods: The study included 73 family caregivers from Romania, aged between 30 and 87 years (M = 57.12, SD = 10.36), the majority being women (75.3%). Latent profile analysis (LPA) was used to identify well-being patterns based on scores obtained on the six dimensions of Ryff’s scale (54 items, adapted for Romania). The selection of models and distinctiveness of profiles were statistically established through various criteria (Akaike Information Criterion (AIC), Bayesian Information Criterion (BIC), and entropy), together with a meaningful interpretation, emphasizing the role of meaning in life, autonomy, and self-acceptance in maintaining caregivers' health, well-being, and resilience.

Results: The LPA analysis identified a four-profile model of well-being, i.e., high (11%), moderate (38%), low (41.1%), and very low (10%), highlighting both the heterogeneity of perceptions and the caregivers’ vulnerabilities and strengths within each profile. Major differences between profiles are primarily driven by the purpose in life and autonomy dimensions, with values progressively decreasing from one profile to the next. The high profile exhibits the highest scores on these dimensions, while the very low profile records the lowest. The most pronounced deficits appear in the very low profile, particularly in self-acceptance and environmental mastery. The high entropy value of the model (0.93) indicates a well-defined solution with significant differences between profiles.

Conclusion: The study highlights variations in well-being among family caregivers of individuals with dementia, making a significant contribution to the identification of distinct latent profiles. A person-centered approach facilitates tailored interventions by clinicians, while the findings provide valuable support both for clinical practitioners and for the development of public health policies.

## Introduction

The increasing prevalence of dementia and other chronic or neurodegenerative conditions in the aging population is leading to a higher demand for long-term care, usually provided by family members [1]. This type of care ensures a better quality of life at a lower cost compared to institutional care [2-4]. The World Health Organization (WHO) prioritizes dementia as a public health issue, proposing a global plan focused on raising awareness, inclusion, risk reduction, patient care, and caregiver support. Special attention is also directed to research, innovation, and the use of information technologies in this field [4].

Family caregivers take on the responsibility for the well-being and daily assistance of those affected by the disease, although often at the cost of their own physical and mental health. Studies in the field show that informal caregiving is associated with an increased risk of psychological stress, burnout, social isolation, and deterioration of the caregivers’ physical and mental health, often turning them into "invisible patients" themselves [5-7]. The burden of caregiving involves the continuous need for patient support, emotional, behavioral, interpersonal, and social challenges, as well as financial difficulties due to the additional costs of care. The negative effects on family caregivers are well documented, but how they experience and manage the challenges of caregiving can vary significantly [8-10].

Public policies regarding dementia focus on the well-being of both patients and their caregivers, whether family or institutional [11]. The concept of well-being includes two components: well-being as a subjective dimension relevant to empirical research in social, psychological, and medical sciences, and quality of life as an objective dimension of interest to economists and sociologists [12,13].

Various psychological and psychosocial models have been developed to assess well-being, exploring different dimensions of it. Among these is the model proposed by Ryff, based on an eudaimonic approach. This model integrates personal development theory, psychosocial theory, and the perspective of positive mental health [14-17]. Ryff’s model emphasizes the positive aspects of life, rather than pathology [6,14,18], and focuses on the individual’s commitment to life’s existential challenges. Thus, well-being is considered a crucial component of personal development, playing a significant role in achieving goals that provide meaning and direction to life [19,20]. It has important functional, emotional, and social implications and is also relevant for the development of public policies [21]. Significant differences between responses on the Ryff’s scale from various groups, including family caregivers of people with dementia, highlight the need for analytical methods that identify distinct response patterns [22-24]. In this regard, the person-centered approach represents an alternative analytical method that prioritizes and analyzes the heterogeneity of individual responses, allowing the identification of distinct profiles (classes) based on similar response patterns. Given that the health and quality of life of family caregivers vary significantly depending on individual characteristics, socio-demographic factors, and cultural contexts, the application of this method contributes to highlighting diversity and creating relevant subject categories.

The identification of subject classes with distinct well-being profiles can be useful for clinicians, the development of targeted intervention programs, and the development of public policies. This approach allows the interpretation of results in relation to subject profiles (classes), facilitating tailored interventions regarding well-being. In this respect, there are studies that have used person-centered analyses to identify family caregiver profiles in the context of other health issues [25-27].

The purpose of the study is to identify subgroups of family caregivers of a family member diagnosed with dementia who share similar response patterns across the six domains of well-being, assessed using Ryff’s scale. The entire study sample includes 73 subjects who have approached the Neurology-Psychiatry Department of the C.F.2 Clinical Hospital in Bucharest. Latent profile analysis (LPA) was used, a statistical method frequently employed in a person-centered approach, to study the heterogeneity of well-being among family caregivers of individuals with dementia. The use of this method for small samples is feasible but requires simple models and well-separated classes. Highlighting similar response patterns in the heterogeneity of well-being among family caregivers may aid in assessing the support needed to reduce caregiving burden.

## Materials and methods

Material

Our study focused on family caregivers of a family member diagnosed with moderate to severe dementia, aged over 30 years, who have accessed medical care at the Neurology-Psychiatry Department of C.F.2 Clinical Hospital in Bucharest between November 2023 and April 2024. Out of the 120 individuals who sought this service, 73 subjects were selected based on inclusion/exclusion criteria. The completion of the questionnaires used in the study was conducted in the presence of the physician conducting the research, within their office at the hospital. The inclusion criteria were a minimum of five years of caregiving experience, age over 30 years, and signed informed consent. Subjects who were providing care to a dementia patient but were not family members, as well as those who did not fully complete the questionnaire items, were excluded from the study.

Methods

The anthropological questionnaire designed for this study collected socio-demographic and economic data, along with information on the living context of both the caregiver and the patient. These included gender, age, place of residence (urban, rural), education level of the primary caregiver (secondary school, high school, higher education), education level of the patient (secondary school, high school, higher education), the presence of family-based caregiving support for the primary contact (single man, woman, within the family) and the declared income of the entire caregiving family.

The well-being of family caregivers in our study was assessed using the 54-item version of Ryff’s scale [15,16,28], adapted for Romania [29], which allowed them to self-assess their perception of well-being. This standardized measure comprises 54 items distributed across six dimensions: autonomy, personal growth, positive relations with others, self-acceptance, purpose in life, and environmental mastery, offering a subjective and comprehensive perspective on one’s own well-being [16,19]. Each dimension is assessed through nine items rated on a six-point Likert scale, ranging from 1 (strongly disagree) to 6 (strongly agree), with 28 items being reverse-scored. Total scores range from 9 to 54 for each dimension, with higher scores reflecting higher well-being. The Ryff’s scale, in its various versions, is widely used in research analyzing the well-being of older adults [30-32] and the impact of caregiving challenges on the well-being of family caregivers of individuals with dementia or other chronic conditions [33,34]. Most studies conducted across different cultures have examined the psychometric properties of the scale [35-37], and the statistical analyses conducted were focused on the variables used to establish relationships between them to identify response patterns.

Data analysis

The main statistical analysis consists of a latent profile analysis (LPA) that separates participants into classes (profiles) that describe the well-being perceptions of family caregivers. The analysis successively tested models from one to 10 profiles. The analysis was implemented using the dedicated *tidyLPA* package developed in the R language, version 4.3.1 (R Foundation for Statistical Computing, Vienna, Austria) [38], as well as with the EXCEL application from the Microsoft Office suite (Microsoft Corporation, Redmond, WA) for graphical visualizations. According to literature [37-39], the optimal model was identified using specific information indices: the Akaike Information Criterion (AIC) and the simple Bayesian Information Criterion (BIC), as well as the sample size-adjusted Bayesian Information Criterion (SABIC). Statistical testing used the bootstrap likelihood ratio test (BLRT), which compares two successive models and is considered more robust than other tests, such as the Vuong-Lo-Mendell-Rubin likelihood ratio test (VLMR-LRT). Additionally, for assessing the accuracy of classification, relative entropy was reported, with values ranging from 0 to 1, with higher values indicating better differentiation.

All participants signed the informed consent, and data were collected after the study protocol was approved by the Ethics Committee of C.F.2 Clinical Hospital (Ref. Number: 1781/06.02.2023).

## Results

Descriptive statistics

The study included 73 family caregivers who maintain contact with the attending physician, of whom 66% (n = 55) are women and 33% (n = 18) are men, with a mean age of 57.12 years (σ = ± 10.363). The majority of family caregivers come from urban areas (67.1%, n = 49). A significant percentage (65.8%, n = 48) receives support from the family, and 30.1% (n = 22) are assisted in caregiving by a woman. In terms of education, the majority of effective caregivers have at least a high school education (80.8%, n = 59). Patients with dementia have a mean age of 78.79 years (σ = ±8.263) and a lower level of education, with the majority having only secondary education (63%, n = 46). Most families have a household income below 1,000 euros (EUR) (78.1%, n = 57) and receive a regular attendance allowance for patient care (Table 1).

**Table 1:** Characteristics of family caregivers in the study sample (N = 73).

Characteristics			
Gender of primary contact caregiver, n (%)	Male	18 (33%)	
	Female	55 (66%)	
Age of primary contact caregiver, n (%)	Mean (SD)	57.12 (10.363)	
	30-57	41 (56.2%)	
	58-87	32 (43.8%)	
Origin of primary contact caregiver, n (%)	Rural	24 (32.9%)	
	Urban	49 (67.1%)	
Family-based caregiving support for the primary contact caregiver, n (%)	Female	22 (30.1%)	
	Family	48 (65.8%)	
	Single person	3 (4.1%)	
Effective caregiver education level, n (%)	Secondary	14 (19.2%)	
	High school	32 (43.8%)	
	Higher education	27 (37%)	
Dementia patient education level, n (%)	Secondary	46 (63%)	
	High school	24 (32.9%)	
	Higher education	3 (4.1)	
Declared income of the entire caregiving family, n (%)	Up to 400 EUR	14 (19.2)	
	400-1000 EUR	43 (58.9%)	
	Equal to or above 1000 EUR	9 (12.3%)	
	Not declared	7 (9.6%)	
Disability allowance	Total	73 (100%)	

The descriptive analysis and statistical correlations reveal a significant degree of heterogeneity in the responses of family caregivers. In particular, they score the highest mean on the dimensions for environmental mastery (M = 38.01) and self-acceptance (M = 37.78), while the lowest scores are recorded for purpose in life (M = 33.14) and personal growth (M = 33.42), indicating specific vulnerabilities in these areas. The increased variability in responses suggests significant differences among participants, which is also reflected in the standard deviations, ranging from SD = 6.367 (autonomy) to SD = 8.699 (environmental mastery). This indicates considerable dispersion in responses, with perceptions of environmental mastery being much more diverse. Additionally, the wide range of scores for the environmental mastery dimension (between 13 and 54) highlights a high variability in caregivers' perceptions, indicating both significant difficulties for some and a high level of well-being for others (Table 2).

**Table 2:** Descriptive statistics for the six domains of Ryff’s well-being (N = 73).

	M	SD	Range	Min.	Max.
SelfAcc	37.78	7.881	33	21	54
PosRel	37.48	7.616	38	16	54
Purpose	33.14	6.721	31	23	54
EnvMastery	38.01	8.699	41	13	54
PersGrowth	33.42	6.627	41	13	54
Autonomy	36.18	6.367	29	23	52

The analysis reveals significant correlations among the dimensions of well-being. Notably, environmental mastery is strongly correlated with both self-acceptance (r = 0.823; p < 0.01) and positive relations with others (r = 0.824; p < 0.01). Additionally, moderate correlations are observed between purpose in life and autonomy (r = 0.498; p < 0.05) and between autonomy and personal growth (r = 0.515; p < 0.05). These findings underscore the interdependence of well-being dimensions and highlight the variability in participants' perceptions and responses (Table 3).

**Table 3:** Pearson correlations between the six domains of psychological well-being. ** The correlation is significant at the 0.01 level (two-tailed).

	SelfAcc	PosRel	PurposeLife	EnvMastery	PersGrowth	Autonomy
SelfAcc	1					
PosRel	0.720^**^	1				
PurposeLife	0.636^**^	0.634^**^	1			
EnvMastery	0.823^**^	0.824^**^	0.670^**^	1		
PersGrowth	0.668^**^	0.793^**^	0.615^**^	0.726^**^	1	
Autonomy	0.604^**^	0.531^**^	0.498^**^	0.539^**^	0.515^**^	1

To identify homogeneous subgroups (profiles) with similar response patterns within our sample of family caregivers, we conducted a latent profile analysis (LPA) using the six dimensions of well-being as input variables. We selected the model with four latent profiles (classes) as optimal.

**Figure 1:**
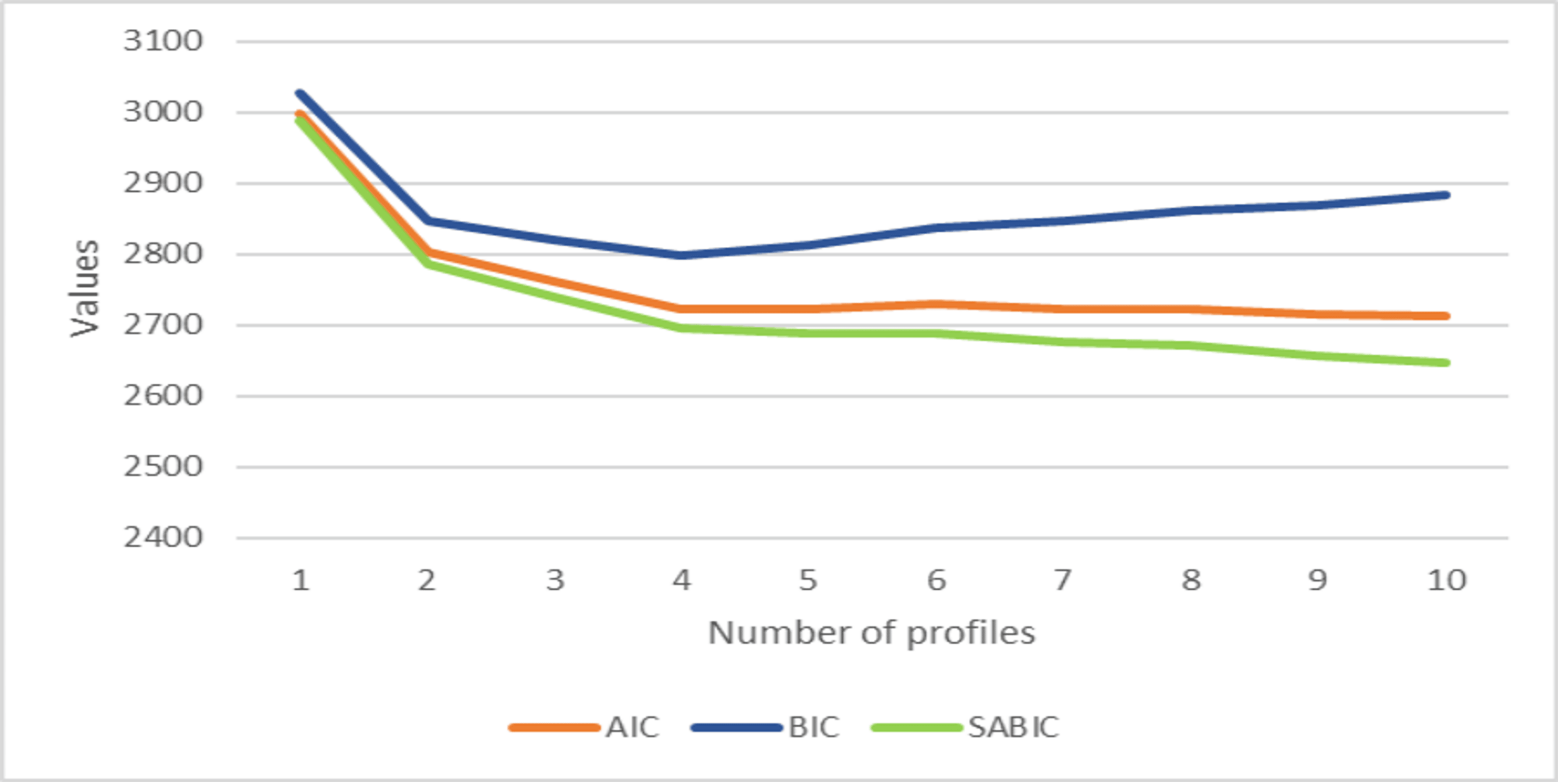
Comparison of the information criteria: Akaike Information Criterion (AIC), Bayesian Information Criterion (BIC), and the sample size-adjusted Bayesian Information Criterion (SABIC).

As we can notice in the plot and table below (Figure 1 and Table 4), the AIC and SABIC indices do not suggest a clear solution, their values following a decreasing tendency with the increase in the number of classes. The BIC index, on the contrary, through its "elbow pattern" minimizes its value for the model with four classes, suggesting it as an optimal solution. This choice is also supported by the solution provided by the *tinyLPA* package, referencing the article [40], which weights several measures for this decision. Additional considerations converge to strengthen the option for the four-class model. One might be seen in the fact that the AIC values, though lacking a global minimum, tend to fluctuate around the local minimum reached for the five-class model, with the AIC value for the four-class model being very close, within this variation range. By further looking at the other measures provided in Table 4, one can notice that the entropy has a high value of over 0.9 up to the five-class model, which means that the four-class model provides a very good differentiation of the profiles. The low BLRT_p probability (0.009) is also a good indicator for the four-class model, radically increasing its value for the next model. Lastly, by performing multiple runs without fixing the seed, the solution of the four-class model indicated by the BIC remained stable. In the end, one of the most important considerations is related to the interpretability of a model, which favors models with fewer classes, as the one in our case, with only four classes.

**Table 4:** Indices for model selection. AIC: Akaike Information Criterion; BIC: Bayesian Information Criterion; SABIC: sample size-adjusted Bayesian Information Criterion; BLRT: bootstrap likelihood ratio test.

Classes	AIC	BIC	SABIC	Entropy	BLRT_p
1	2999.16	3026.64	2988.83	1.00	
2	2802.46	2845.98	2786.11	0.94	0.01
3	2761.56	2821.11	2739.19	0.93	0.01
4	2723.56	2799.15	2695.16	0.93	0.01
5	2721.73	2813.35	2687.31	0.91	0.16
6	2728.78	2836.44	2688.34	0.87	0.88
7	2722.45	2846.13	2675.98	0.88	0.05
8	2722.93	2862.65	2670.44	0.90	0.26
9	2714.21	2869.97	2655.70	0.92	0.02
10	2711.59	2883.37	2647.05	0.94	0.18

In the four-profile model, profile 3 is the most prevalent, comprising 41% (n = 30) of participants, followed by profile 2 at 38% (n = 28). Together, these two profiles account for 79.46% of the family caregivers. Profiles 1 and 4 are less common, with profile 1 representing 10.96% (n = 8) and profile 4 representing 9.59% (n = 7) of participants, indicating that these may be marginal or specific groups.

**Figure 2:**
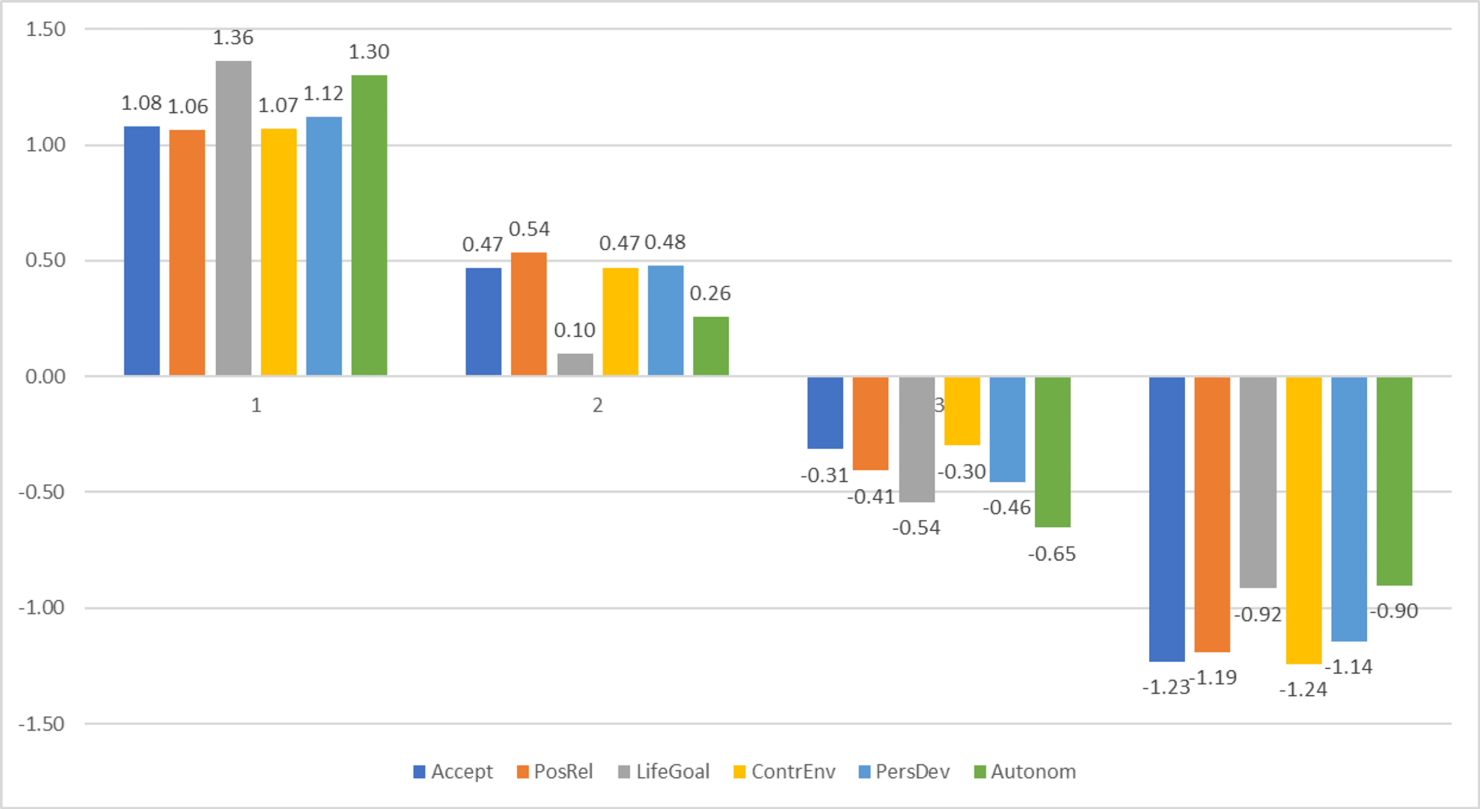
Plot of standardized z-score profiles showing four latent well-being profiles. Profile 1 represents caregivers with the highest well-being scores, while profile 4 captures the lowest levels of psychological well-being across all six dimensions.

The four-profile model, as illustrated in Figure 2, utilized z-scores to highlight variations in well-being dimensions relative to their means. Profiles 1 and 2, with positive z-scores, indicate higher well-being levels and are categorized as "High" and "Moderate" well-being, respectively. Conversely, profiles 3 and 4, with negative z-scores, suggest challenges in patient care and are labeled as "Low" and "Very Low" well-being. Profile 1 is characterized by consistently positive z-scores across all dimensions of psychological well-being, reflecting a high-functioning subgroup of caregivers. In contrast, profile 4 displays negative z-scores on all six dimensions, indicating the most vulnerable profile. The most pronounced differences in z-scores between profiles are observed in the following dimensions: self-acceptance (2.31), purpose in life (2.28), and autonomy (2.20), which most distinctly differentiate participants across profiles (Figure 2).

Profile 1, labeled "High Well-Being," represents a marginal group, comprising 10.96% (n = 8) of the subjects. This profile is characterized by positive z-scores exceeding 1 across all dimensions, indicating a significantly higher level of well-being compared to the overall sample mean. The highest values are recorded for the dimensions of purpose in life (z = 1.36) and autonomy (z = 1.30).

Profile 2, labeled "Moderate Well-Being," comprises 38.36% (n = 28) of the subjects, making it one of the majority groups. This profile is characterized by slightly positive z-scores, close to the sample mean. The highest values are observed in self-acceptance (z = 0.47), environmental mastery (z = 0.47), positive relations with others (z = 0.54), and personal growth (z = 0.48), while the lowest are in purpose in life (z = 0.10) and autonomy (z = 0.26).

Profile 3, labeled "Low Well-Being," comprises 41.10% (n = 30) of the family caregivers, making it the majority group. This profile is characterized by negative z-scores, close to zero, across all dimensions, placing it below the overall sample mean. The most notable deficits are observed in autonomy (z = -0.65) and purpose in life (z = -0.54). Smaller deficits are noted in self-acceptance (z = -0.31) and environmental mastery (z = -0.30).

Profile 4, labeled "Very Low Well-Being," includes 10% (n = 7) of the sample and is also a marginal group of subjects, defined by significantly negative z-scores across all analyzed dimensions. The lowest z-values are found in environmental mastery (z = -1.24) and self-acceptance (z = -1.23).

## Discussion

This study investigated a sample of family caregivers in Romania who provide care for individuals with moderate to severe dementia and who sought services at the Neurology-Psychiatry Department of the C.F.2 Clinical Hospital in Bucharest. The LPA identified four distinct well-being profiles among family caregivers: "High Well-Being," "Moderate Well-Being," "Low Well-Being," and "Very Low Well-Being." The key dimensions that contributed to the differences between profiles, as revealed by the z-score analysis, were autonomy, purpose in life, and self-acceptance.

To our knowledge, no studies have employed LPA to examine the well-being of family caregivers of individuals with dementia using Ryff’s model. However, research in this area has identified and analyzed significant variations among caregiver subgroups regarding their well-being using other statistical methods, such as confirmatory factor analysis, multiple regression, and structural equation modeling [16,41,42]. For our study sample, LPA proved useful in exploring variations in well-being, and the four-class model was found to be optimal based on statistical and interpretability criteria. The entropy measure remained high (>0.9) up to the five-class model, indicating a clear distinction between profiles. However, these findings may be specific to the analyzed population, suggesting that the well-being of family caregivers varies considerably, being influenced by both caregiving burden and personal resources.

The “high well-being” profile consists of a small group of participants characterized by elevated scores in the autonomy and purpose in life dimensions, suggesting that these family caregivers find meaning and direction in their lives. They maintain an optimistic outlook, demonstrating a balance across well-being dimensions and strong resilience. Both men and women from rural and urban areas, aged between 40 and 60 years (M = 54.38, SD = 5.476), maintain contact with the attending physician. Caregiving is provided by the entire family, and their educational level is higher than that of the care recipient, including high school and higher education. The family's income is low, indicating limited resources in the caregiving context.

The "moderate well-being" profile represents one of the majority groups, with a moderate level of well-being, slightly above the sample average. Caregivers in this group manage to balance caregiving responsibilities and supportive relationships but face challenges in achieving autonomy or personal goals. Contact with the attending physician is predominantly maintained by women (75%) from urban areas (67.1%), aged between 34 and 76 years (M = 55.68, SD = 10.281). Caregiving is provided either by the entire family (67.9%) or a single individual (32.1%). The majority have a high school education or higher (92.9%), and the family incomes are low.

The "low well-being" profile represents one of the largest groups, characterized by low levels of well-being, limited autonomy, and difficulties in maintaining or achieving personal goals. Participants often feel overwhelmed by their caregiving responsibilities. Both men and women in this profile maintain contact with the attending physician, with the majority living in urban areas (60%) and ranging in age from 30 to 87 years (M = 58.07, SD = 11.814). Caregiving is provided either by the entire family (63.3%) or by an individual caregiver (36.7%). Most participants have completed high school or hold a higher education degree (63.3%), while family income remains low, reflecting financial constraints.

The "very low well-being" profile encompasses a small group of participants who perceive their well-being as significantly below the study average. Their reduced sense of control over their external environment places them in a state of pronounced psychosocial and economic vulnerability. Contact with the attending physician is maintained exclusively by women from rural areas, aged between 53 and 73 years (M = 62, SD = 7.165). Care responsibilities are shared within the family unit, with all participants holding at least a high school diploma (100%). Their low family household income within this group likely compounds their overall vulnerability.

A comparison of the four well-being profiles among family caregivers reveals significant differences in how they perceive life and manage caregiving responsibilities. The purpose in life and autonomy dimensions emerge as key factors in distinguishing these profiles. Caregivers in the high well-being profile exhibit the highest scores in these areas, underscoring the importance of having clear goals and maintaining autonomy. In contrast, the moderate well-being profile aligns closely with the sample average, while the low well-being and very low well-being profiles demonstrate substantial difficulties, reflected in their below-average scores.

A progressive decline in self-acceptance is observed across profiles with lower well-being, indicating increasing vulnerability among participants. Similarly, environmental mastery and positive relations with others reach their peak in the high well-being group and their lowest levels in the very low well-being group. Notably, caregivers in the moderate well-being profile sustain a relative balance through higher scores in these dimensions, whereas those in the low well-being group present consistently low values. These findings highlight the critical role of environmental mastery and supportive interpersonal relationships in shaping and differentiating well-being among family caregivers.

The pivotal roles of purpose in life and autonomy as predictors of well-being have been consistently underscored in prior research. Within his conceptual framework of well-being, Ryff identified autonomy and purpose in life as core dimensions essential for achieving elevated levels of well-being [15], fostering both psychological resilience and overall life satisfaction [34,43]. Empirical evidence further supports the connection between well-being and physical health, highlighting the regulatory influence of these dimensions on key physiological systems [34,44]. Similarly, self-acceptance, reflecting one’s attitude toward oneself and the acknowledgment of personal limitations, has been shown to enhance resilience to external evaluations, thereby contributing to the preservation of both health and well-being [34,45-47].

By utilizing LPA, a person-centered methodology, alongside Ryff's model, this study identified distinct well-being profiles among family caregivers of individuals with dementia, highlighting the heterogeneity of self-perception and the diversity of their experiences. The results emphasize the usefulness of LPA in identifying specific combinations of well-being dimensions, analyzing the prevalence of profiles, and tailoring interventions to the particular needs of each subgroup.

For example, for those within the very low well-being profile, interventions should prioritize enhancing self-acceptance and environmental mastery, as these individuals may benefit significantly from emotional support and stability. In contrast, for those categorized under the moderate well-being profile, promoting the development of personal goals and autonomy becomes a priority to improve their responsiveness to interventions aimed at strengthening self-determination. Similarly, for caregivers in the low well-being profile, targeted efforts to enhance interpersonal relationships are critical, given the detrimental effects of social isolation on health and overall well-being.

Limitations

Although the four-class solution proved to be optimal, enabling the analysis and interpretation of results, the small sample size limits the exploration of differences between the identified latent profiles based on socio-demographic characteristics or other relevant variables outside the classification model. This constraint reduces the study’s capacity to establish causal inferences. Furthermore, as the research was conducted exclusively in a single medical unit in Romania, the findings cannot yet be generalized at either the national or international level. Nevertheless, the use of a person-centered approach successfully distinguished distinct latent profiles among participants, offering a valuable foundation for more targeted interventions.

## Conclusions

This study provides a valuable framework for developing tailored medical interventions and public health policies aimed at enhancing the quality of life for family caregivers of individuals with dementia. The results offer an understanding of the variability in family caregiving experiences for patients. By capturing the variability in caregiving experiences, the identification of four distinct latent profiles, derived through a person-centered approach, highlights both the internal resources that can be strengthened and the specific challenges faced by each group. These insights enable the design of targeted interventions and support strategies aimed at alleviating caregiver burden and promoting overall well-being.
